# Supplementary material for: Target Lines for in Planta Gene Stacking in Japonica Rice
Source: Int J Mol Sci. 2022 Aug 20;23(16):9385. doi: 10.3390/ijms23169385 (PMC9409015; doi:10.3390/ijms23169385)
Supplement: Supplementary file 1 [file ijms-23-09385-s001.zip › Supplementary info/Li et al SupFigures S1-S6 20220815.pdf]

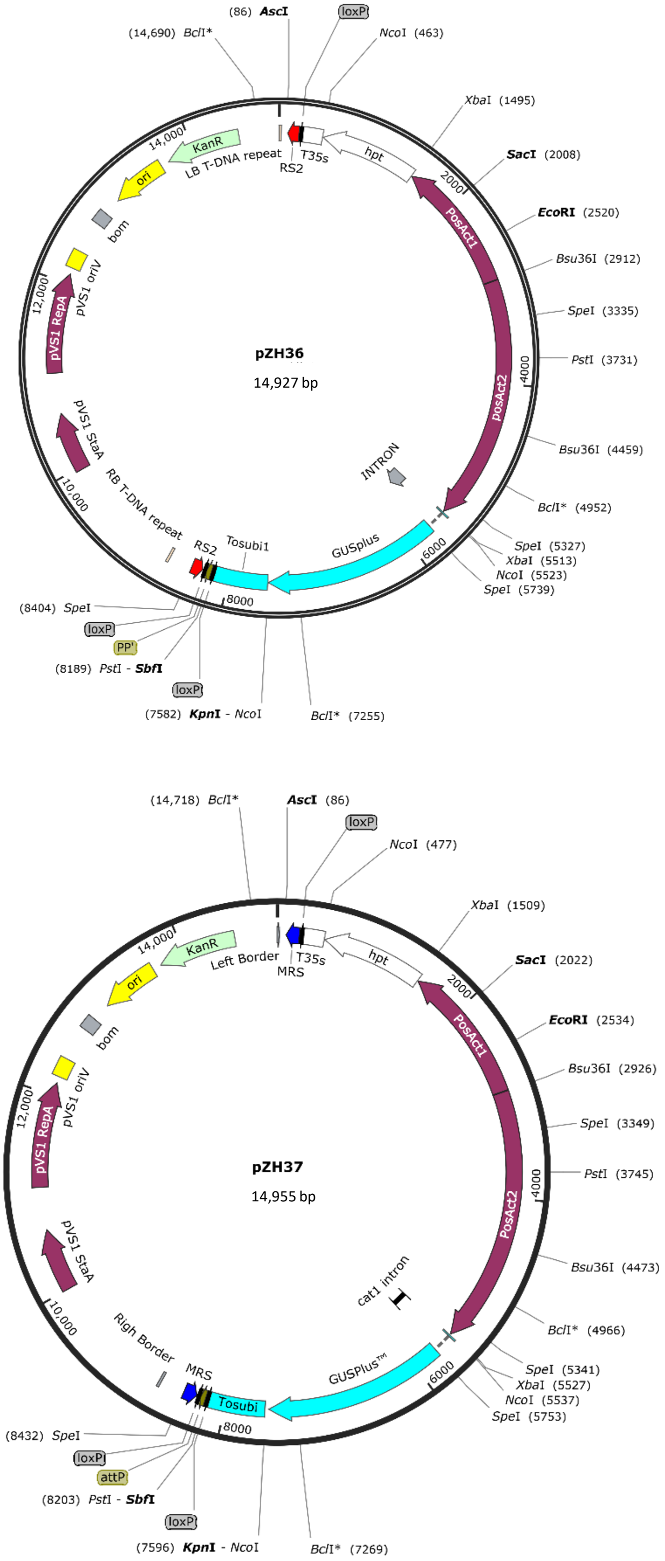

Figure S1. Map of pZH36 and pZH37.

Figure S2

(A)

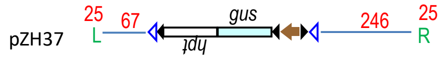


```
              5187268                   5187244                                                                                  5187205                    5187181
Chr2:5187268  TCCAGGGAAGAAAAATGCACTAGAAGATTATATTCTACTCCTTTTCAGATGCCGTATGCATCG-----------------------------------GTGCTCTCTATTTCCCGACGAGATA
131           TCCAGGGAAGAAAAATGCACTAGAACACATTGCGGACGTTTTTAATGTACT (full T-DNA not shown) TTCAGTTTAAACTATCAGTGTTTGAGTGCTCTCTATTTCCCGACGAGATA

               27877788                 27877812                                                                                 27877843                   27877867
Chr5:27877788  ACAGTACGCTGAAATATACTGACGCCGTAGGGGACAGCGTCCAGGACGATAATAA-------------------------------------------AAAAATTCTACGAGATCTGGATCGC
284            ACAGTACGCTGAAATATACTGACGCTTAGACAACTTAATAACACATTGCG (full T-DNA not shown) CTTCAGTTTAAACTATCAGTGTTTGAAAAAATTCTACGAGATCTGGATCGC

               32100617                 32100641                                                                                 32100689                   32100713
Chr1:32100617  TACATCTGCTTAGCTAACTGCTTGCTGCATGCATGCAAACCCTAGCTACAGGGCTATGCAGACACAGTGCAC------------------------GCAGAGAGAGAGAGAGAGAGAGAGA
325            TACATCTGCTTAGCTAACTGCTTGCGGACGTTTAGACAACTTAATAACAC (full T-DNA not shown) TTCAGTTTAAACTATCAGTGTTTGAGCAGAGAGAGAGAGAGAGAGAGAGA

               35913990                 35913966                                                                                 35913934                   35913910
Chr1:35913990  GGCTGACGACGATCGCTCGCCCGTGCGTGTCGCGCTCGCGGTCGATCGAGTACGTA-----------------------------------------CGCCAATAATAATGCGCGCGGTGTG
537            GGCTGACGACGATCGCTCGCCCGTGTGTAAACAAATTGACGCTTAGACA (full T-DNA not shown) TTCAGTTTAAACTATCAGTGTTTGACGCCAATAATAATGCGCGCGGTGTG
```

(B)

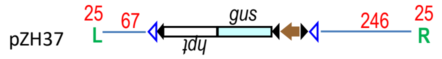


```
               16669183                 16669159                                                                                 16669154                   16669130
Chr8:16669183  ATTTGAAGTTCTCTTACCTGGGTTTGTCG--------------------------------------------------------------------GTCTCTACTTGTAGGACTGCGAGGG
281            ATTTGAAGTTCTCTTACCTGGGTTTTAAGAACTTTTATATTGTGGTGTAAAC  (full T-DNA not shown) TAGTTTAAACTATCAGCCCTCGCAGTCTCTACTTGTAGGACTGCGAGGG

               27601532                     27601562                                                                             27601606                   27601630
Chr5:27601532  AAAGGGGGGGGCTTCCGCCGACCGGGCCCGTCCCCCCCTGCCCGTGCTGATGGGCCGGGGCTCCGATTAAAGGA------------------------CCATATTTGCACATACCACCCTACA
367            AAAGGGGGGGGCTTCCGCCGACCGGGCCCGTAAATTATAAATCTTAAATA (full T-DNA not shown) TTCAGTTTAAACTATCAGTGTTTGACCATATTTGCACATACCACCCTACA
                                           •••

               9639384                  9639408                                                                                  9639426                    9639450
Chr1:9639384   GCGCTCACGGTAGACGTGACAACGACACTTTCGCCGTATGGA-------------------------------------------------------GCGGTGGAGCCCACCCACCCATCCT
766            GCGCTCACGGTAGACGTGACAACGATAAACAAATTGACGCTTAGACAACT (full T-DNA not shown) TTCAGTTTAAACTATCAGTGTTTGAGCGGTGGAGCCCACCCACCCATCCT
```

Figure S2. Flanking DNA of the target line insertion sites. **(A)** Flanking DNA for pZH37-derived insertion sites. **(B)** Flanking DNA for pZH36-derived insertion sites. Chromosome position numbers according to http://rapdb.dna.affrc.go.jp/ Database. Due to orienting the target construct as depicted in Figure 1A (left border left, right border right), the chromosome position numbers may increase or decrease from left to right. Purple lettering indicates deleted host nucleotide bases. Pink lettering indicates insertion of DNA that does not originate from the insertion site or from the vector. Green lettering indicates sequences corresponding to the T-DNA borders. Blue lettering indicates sequences corresponding to pZH36 or pZH37 vector sequences. Red lettering indicates sequences corresponding to recombination site *RS2*. Black dot indicates the overlaps with the host DNA. Dot below nucleotide indicates the same nucleotide whether from host or transgenic DNA

Figure S3

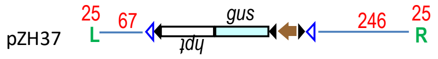


```
             Left border                                                                                  MRS
PZH37        TGGCAGGATATATTGTGGTGTAAACAAATTGACGCTTAGACAACTTAATAACACATTGCGGACGTTTTTAATGTACTGAATTAAGGCGCGCCCTCATATGTGGGCGTGAGGCTACAGCGC
131          ---------------------------------------------------CACATTGCGGACGTTTTTAATGTACTGAATTAAGGCGCGCCCTCATATGTGGGCGTGAGGCTACAGCGC
284          -----------------------------------TTAGACAACTTAATAACACATTGCGGACGTTTTTAATGTACTGAATTAAGGCGCGCCCTCATATGTGGGCGTGAGGCTACAGCGC
325          -----------------------------GGACGTTTAGACAACTTAATAACACATTGCGGACGTTTTTAATGTACTGAATTAAGGCGCGCCCTCATATGTGGGCGTGAGGCTACAGCGC
537          -----------------GTGTAAACAAATTGACGCTTAGACAACTTAATAACACATTGCGGACGTTTTTAATGTACTGAATTAAGGCGCGCCCTCATATGTGGGCGTGAGGCTACAGCGC
                             •

                                                                                                                            lox
PZH37        GAGGCGCATTAAGGTCGGGAAAATGCGCTAGGCGCATTTAAATTGCGTATTGCTGTAATGCGCCATGCCGGCTAGACTAGGCCCAAATGGGTATACCCAATTTGATTAATTAAATAACTT
131          GAGGCGCATTAAGGTCGGGAAAATGCGCTAGGCGCATTTAAATTGCGTATTGCTGTAATGCGCCATGCCGGCTAGACTAGGCCCAAATGGGTATACCCAATTTGATTAATTAAATAACTT
284          GAGGCGCATTAAGGTCGGGAAAATGCGCTAGGCGCATTTAAATTGCGTATTGCTGTAATGCGCCATGCCGGCTAGACTAGGCCCAAATGGGTATACCCAATTTGATTAATTAAATAACTT
325          GAGGCGCATTAAGGTCGGGAAAATGCGCTAGGCGCATTTAAATTGCGTATTGCTGTAATGCGCCATGCCGGCTAGACTAGGCCCAAATGGGTATACCCAATTTGATTAATTAAATAACTT
537          GAGGCGCATTAAGGTCGGGAAAATGCGCTAGGCGCATTTAAATTGCGTATTGCTGTAATGCGCCATGCCGGCTAGACTAGGCCCAAATGGGTATACCCAATTTGATTAATTAAATAACTT

PZH37        CGTATAGCATACATTATACGAAGTTAT
131          CGTATAGCATACATTATACGAAGTTAT
284          CGTATAGCATACATTATACGAAGTTAT
325          CGTATAGCATACATTATACGAAGTTAT
537          CGTATAGCATACATTATACGAAGTTAT

             lox                                    attP                                             lox
PZH37        ATAACTTCGTATAGCATACATTATACGAAGTTATCCTGCAGGGGGTTTGTACCGTACACCACTGAGACCGCGGTGGTTGACCAGACAAACCACTGATAACTTCGTATAATGTATGCTA
131          ATAACTTCGTATAGCATACATTATACGAAGTTATCCTGCAGGGGGTTTGTACCGTACACCACTGAGACCGCGGTGGTTGACCAGACAAACCACTGATAACTTCGTATAATGTATGCTA
284          ATAACTTCGTATAGCATACATTATACGAAGTTATCCTGCAGGGGGTTTGTACCGTACACCACTGAGACCGCGGTGGTTGACCAGACAAACCACTGATAACTTCGTATAATGTATGCTA
325          ATAACTTCGTATAGCATACATTATACGAAGTTATCCTGCAGGGGGTTTGTACCGTACACCACTGAGACCGCGGTGGTTGACCAGACAAACCACTGATAACTTCGTATAATGTATGCTA
537          ATAACTTCGTATAGCATACATTATACGAAGTTATCCTGCAGGGGGTTTGTACCGTACACCACTGAGACCGCGGTGGTTGACCAGACAAACCACTGATAACTTCGTATAATGTATGCTA
                               MRS
PZH37        TACGAAGTTATCCCGGGCTCATATGTGGGCGTGAGGCTACAGCGCGAGGCGCATTAAGGTCGGGAAAATGCGCTAGGCGCATTTAAATTGCGTATTGCTGTAATGCGCCATGCCGGCTAG
131          TACGAAGTTATCCCGGGCTCATATGTGGGCGTGAGGCTACAGCGCGAGGCGCATTAAGGTCGGGAAAATGCGCTAGGCGCATTTAAATTGCGTATTGCTGTAATGCGCCATGCCGGCTAG
284          TACGAAGTTATCCCGGGCTCATATGTGGGCGTGAGGCTACAGCGCGAGGCGCATTAAGGTCGGGAAAATGCGCTAGGCGCATTTAAATTGCGTATTGCTGTAATGCGCCATGCCGGCTAG
325          TACGAAGTTATCCCGGGCTCATATGTGGGCGTGAGGCTACAGCGCGAGGCGCATTAAGGTCGGGAAAATGCGCTAGGCGCATTTAAATTGCGTATTGCTGTAATGCGCCATGCCGGCTAG
537          TACGAAGTTATCCCGGGCTCATATGTGGGCGTGAGGCTACAGCGCGAGGCGCATTAAGGTCGGGAAAATGCGCTAGGCGCATTTAAATTGCGTATTGCTGTAATGCGCCATGCCGGCTAG
                                                  LacZ alpha
PZH37        ACTAGGCCCAAATGGGTATACCCAATTTGAACTAGTATGCAGGCATGCAAGCTTGGCACTGGCCGTCGTTTTACAACGTCGTGACTGGGAAAACCCTGGCGTTACCCAACTTAATCGCCT
131          ACTAGGCCCAAATGGGTATACCCAATTTGAACTAGTATGCAGGCATGCAAGCTTGGCACTGGCCGTCGTTTTACAACGTCGTGACTGGGAAAACCCTGGCGTTACCCAACTTAATCGCCT
284          ACTAGGCCCAAATGGGTATACCCAATTTGAACTAGTATGCAGGCATGCAAGCTTGGCACTGGCCGTCGTTTTACAACGTCGTGACTGGGAAAACCCTGGCGTTACCCAACTTAATCGCCT
325          ACTAGGCCCAAATGGGTATACCCAATTTGAACTAGTATGCAGGCATGCAAGCTTGGCACTGGCCGTCGTTTTACAACGTCGTGACTGGGAAAACCCTGGCGTTACCCAACTTAATCGCCT
537          ACTAGGCCCAAATGGGTATACCCAATTTGAACTAGTATGCAGGCATGCAAGCTTGGCACTGGCCGTCGTTTTACAACGTCGTGACTGGGAAAACCCTGGCGTTACCCAACTTAATCGCCT

PZH37        TGCAGCACATCCCCCTTTCGCCAGCTGGCGTAATAGCGAAGAGGCCCGCACCGATCGCCCTTCCCAACAGTTGCGCAGCCTGAATGGCGAATGCTAGAGCAGCTTGAGCTTGGATCAGAT
131          TGCAGCACATCCCCCTTTCGCCAGCTGGCGTAATAGCGAAGAGGCCCGCACCGATCGCCCTTCCCAACAGTTGCGCAGCCTGAATGGCGAATGCTAGAGCAGCTTGAGCTTGGATCAGAT
284          TGCAGCACATCCCCCTTTCGCCAGCTGGCGTAATAGCGAAGAGGCCCGCACCGATCGCCCTTCCCAACAGTTGCGCAGCCTGAATGGCGAATGCTAGAGCAGCTTGAGCTTGGATCAGAT
325          TGCAGCACATCCCCCTTTCGCCAGCTGGCGTAATAGCGAAGAGGCCCGCACCGATCGCCCTTCCCAACAGTTGCGCAGCCTGAATGGCGAATGCTAGAGCAGCTTGAGCTTGGATCAGAT
537          TGCAGCACATCCCCCTTTCGCCAGCTGGCGTAATAGCGAAGAGGCCCGCACCGATCGCCCTTCCCAACAGTTGCGCAGCCTGAATGGCGAATGCTAGAGCAGCTTGAGCTTGGATCAGAT
                                               Right border
PZH37        TGTCGTTTCCCGCCTTCAGTTTAAACTATCAGTGTTTGACAGGATATATTGGCGGGTAAAC
131          TGTCGTTTCCCGCCTTCAGTTTAAACTATCAGTGTTTGA----------------------
284          TGTCGTTTCCCGCCTTCAGTTTAAACTATCAGTGTTTGA----------------------
325          TGTCGTTTCCCGCCTTCAGTTTAAACTATCAGTGTTTGA----------------------
537          TGTCGTTTCCCGCCTTCAGTTTAAACTATCAGTGTTTGA----------------------
```

Figure S3. pZH37-derived target site sequences from left and right end of the T-DNA to the innermost lox sites. Gray bars show sequence rangeto pZH37 vector sequences. Blue, bold black and brown lettering indicates sequences corresponding to. Green lettering indicates sequences corresponding to the T-DNA borders. Black lettering indicates sequences corresponding recombination site *MRS, lox* and *attP* sites, respectively. Right border vector sequence contains the *E. coli lacZ* alpha fragment coding sequence. Black dot below nucleotide indicates the same nucleotide whether from host or T-DNA left border DNA.

Figure S4

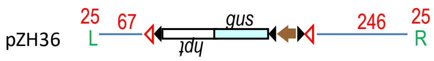


```
                Left border                                                                                    RS2
PZH36   --TGGCAGGATATATTGTGGTGTAAACAAATTGACGCTTAGACAACTTAATAACACATTGCGGACGTTTTTAATGTACTGAATTAAGGCGCGCCCGTAAATTATAAATCTTAAATATCAA
281     TAAGAACTTTTATATTGTGGTGTAAACAAATTGACGCTTAGACAACTTAATAACACATTGCGGACGTTTTTAATGTACTGAATTAAGGCGCGCCCGTAAATTATAAATCTTAAATATCAA
367     -------------------------------------------------------------------------------------------GCCCGTAAATTATAAATCTTAAATATCAA
                                                                                                      •••
766     ----------------------TAAACAAATTGACGCTTAGACAACTTAATAACACATTGCGGACGTTTTTAATGTACTGAATTAAGGCGCGCCCGTAAATTATAAATCTTAAATATCAA

                                                                                                              lox
PZH36   AGTTACATGTTATATATGGTTAAAAATCATTTAAATGTTACATAGTTTTAAGAACTTTTATATTGTAACTTTAGGGTATACCCCAAAGTAACGTTAATTAAATAACTTCGTATAGCATAC
281     AGTTACATGTTATATATGGTTAAAAATCATTTAAATGTTACATAGTTTTAAGAACTTTTATATTGTAACTTTAGGGTATACCCCAAAGTAACGTTAATTAAATAACTTCGTATAGCATAC
367     AGTTACATGTTATATATGGTTAAAAATCATTTAAATGTTACATAGTTTTAAGAACTTTTATATTGTAACTTTAGGGTATACCCCAAAGTAACGTTAATTAAATAACTTCGTATAGCATAC
766     AGTTACATGTTATATATGGTTAAAAATCATTTAAATGTTACATAGTTTTAAGAACTTTTATATTGTAACTTTAGGGTATACCCCAAAGTAACGTTAATTAAATAACTTCGTATAGCATAC

PZH36   ATTATACGAAGTTAT
281     ATTATACGAAGTTAT
367     ATTATACGAAGTTAT
766     ATTATACGAAGTTAT

        lox                                   attP                                                        lox
PZH36   ATAACTTCGTATAGCATACATTATACGAAGTTATCCTGCAGGGGGTTTGTACCGTACACCACTGAGACCGCGGTGGTTGACCAGACAAACCACTGATAACTTCGTATAATGTATGCTATA
281     ATAACTTCGTATAGCATACATTATACGAAGTTATCCTGCAGGGGGTTTGTACCGTACACCACTGAGACCGCGGTGGTTGACCAGACAAACCACTGATAACTTCGTATAATGTATGCTATA
367     ATAACTTCGTATAGCATACATTATACGAAGTTATCCTGCAGGGGGTTTGTACCGTACACCACTGAGACCGCGGTGGTTGACCAGACAAACCACTGATAACTTCGTATAATGTATGCTATA
766     ATAACTTCGTATAGCATACATTATACGAAGTTATCCTGCAGGGGGTTTGTACCGTACACCACTGAGACCGCGGTGGTTGACCAGACAAACCACTGATAACTTCGTATAATGTATGCTATA

                     RS2
PZH36   CGAAGTTATCCCGGGCGTAAATTATAAATCTTAAATATCAAAGTTACATGTTATATATGGTTAAAAATCATTTAAATGTTACATAGTTTTAAGAACTTTTATATTGTAACTTTAGGGTAT
281     CGAAGTTATCCCGGGCGTAAATTATAAATCTTAAATATCAAAGTTACATGTTATATATGGTTAAAAATCATTTAAATGTTACATAGTTTTAAGAACTTTTATATTGTAACTTTAGGGTAT
367     CGAAGTTATCCCGGGCGTAAATTATAAATCTTAAATATCAAAGTTACATGTTATATATGGTTAAAAATCATTTAAATGTTACATAGTTTTAAGAACTTTTATATTGTAACTTTAGGGTAT
766     CGAAGTTATCCCGGGCGTAAATTATAAATCTTAAATATCAAAGTTACATGTTATATATGGTTAAAAATCATTTAAATGTTACATAGTTTTAAGAACTTTTATATTGTAACTTTAGGGTAT

                   lacZ alpha
PZH36   ACCCCAAAGTAACGACTAGTATGCAGGCATGCAAGCTTGGCACTGGCCGTCGTTTTACAACGTCGTGACTGGGAAAACCCTGGCGTTACCCAACTTAATCGCCTTGCAGCACATCCCCCT
281     ACCCCAAAGTAACGACTAGTATGCAGGCATGCAAGCTTGGCACTGGCCGTCGTTTTACAACGTCGTGACTGGGAAAACCCTGGCGTTACCCAACTTAATCGCCTTGCAGCACATCCCCCT
367     ACCCCAAAGTAACGACTAGTATGCAGGCATGCAAGCTTGGCACTGGCCGTCGTTTTACAACGTCGTGACTGGGAAAACCCTGGCGTTACCCAACTTAATCGCCTTGCAGCACATCCCCCT
766     ACCCCAAAGTAACGACTAGTATGCAGGCATGCAAGCTTGGCACTGGCCGTCGTTTTACAACGTCGTGACTGGGAAAACCCTGGCGTTACCCAACTTAATCGCCTTGCAGCACATCCCCCT

PZH36   TTCGCCAGCTGGCGTAATAGCGAAGAGGCCCGCACCGATCGCCCTTCCCAACAGTTGCGCAGCCTGAATGGCGAATGCTAGAGCAGCTTGAGCTTGGATCAGATTGTCGTTTCCCGCCTT
281     TTCGCCAGCTGGCGTAATAGCGAAGAGGCCCGCACCGATCGCCCTTCCCAACAGTTGCGCAGCCTGAATGGCGAATGCTAGAGCAGCTTGAGCTTGGATCAGATTGTCGTTTCCCGCCTT
367     TTCGCCAGCTGGCGTAATAGCGAAGAGGCCCGCACCGATCGCCCTTCCCAACAGTTGCGCAGCCTGAATGGCGAATGCTAGAGCAGCTTGAGCTTGGATCAGATTGTCGTTTCCCGCCTT
766     TTCGCCAGCTGGCGTAATAGCGAAGAGGCCCGCACCGATCGCCCTTCCCAACAGTTGCGCAGCCTGAATGGCGAATGCTAGAGCAGCTTGAGCTTGGATCAGATTGTCGTTTCCCGCCTT

                             Right border
PZH36   CAGTTTAAACTATCAGTGTTTGACAGGATATATTGGCGGGTAAAC
281     CAGTTTAAACTATCAGTGTTTGATAGTTTAAACTATCAGCCCTCGCA
367     CAGTTTAAACTATCAGTGTTTGA-----------------------
766     CAGTTTAAACTATCAGTGTTTGA-----------------------
```

Figure S4. pZH36-derived target site sequences from left and right boundaries of the T-DNA to the innermost *lox* sites. Green lettering indicates sequences corresponding to T-DNA borders. Pink lettering indicates insertion of DNA not from the insertion site or the vector. Black lettering indicates sequences corresponding to pZH36 vector sequences. Red, bold black and brown lettering indicates sequences corresponding to recombination site *RS2*, *lox* and *attP* sites, respectively. Right border vector sequence contains the *E. coli lacZ* alpha fragment coding sequence. Black dot below nucleotide indicates the same nucleotide whether from host or transgenic DNA.

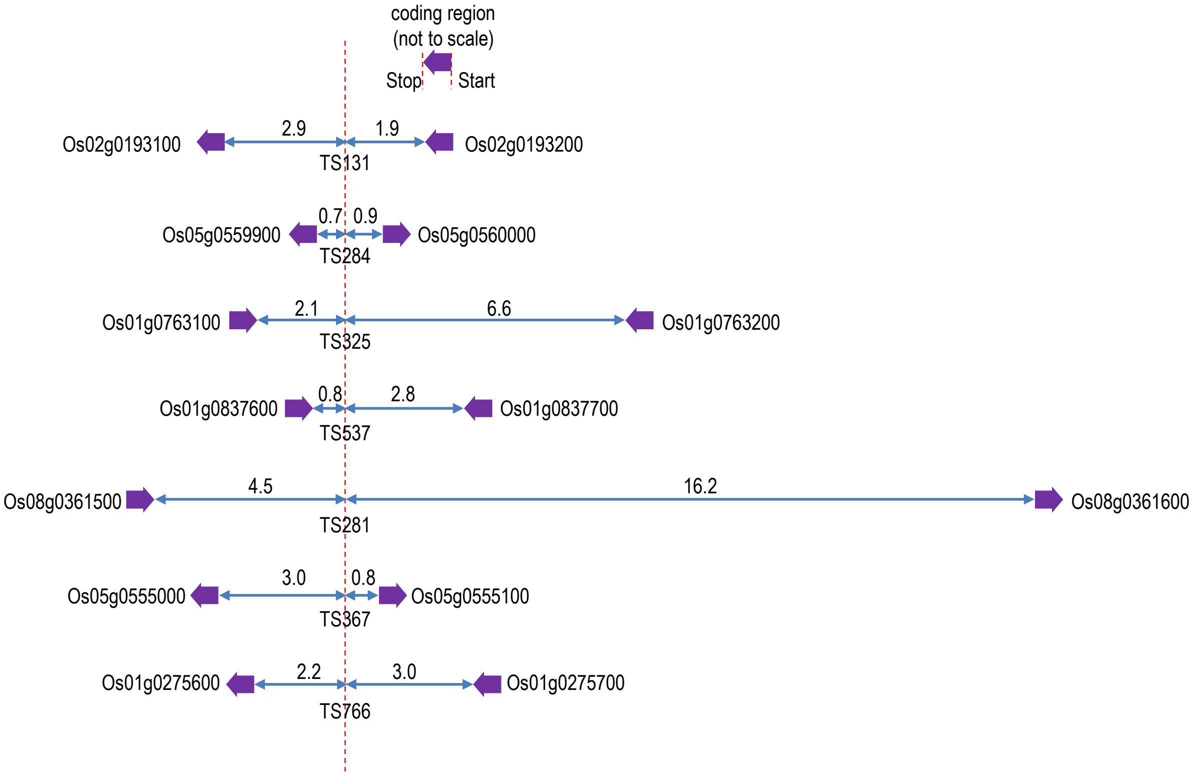

Figure S5 Rice target sites and nearest coding regions.

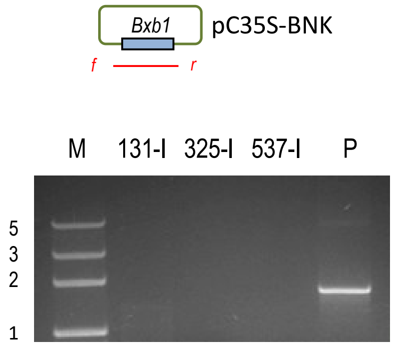

Figure S6. Lack of PCR detection of Bxb1 integrase DNA in T2 integrants 131-I, 325-I and 537-I. Primers *f* and *r* correspond to Bxb1 integrase coding region. P: positive control with pC35S-BNK DNA diluted to 1 copy per diploid rice genome; M: molecular markers in kb.
