# Supplementary material for: Target Lines for in Planta Gene Stacking in Japonica Rice
Source: Int J Mol Sci. 2022 Aug 20;23(16):9385. doi: 10.3390/ijms23169385 (PMC9409015; doi:10.3390/ijms23169385)
Supplement: Supplementary file 1 [file ijms-23-09385-s001.zip › Supplementary info/Li et al. rice SupInfo TableS1-20220815.pdf]

Table S1. Summary of screening for rice target lines

Screening	No. of plants for pZH36	No. of plants for pZH37	Total
hygromycin-resistant	1717	2236	3953
gusplus PCR	1566	1570	3136
1st qPCR (*gus*)	844	600	1444
2nd qPCR (*hp*t)	307	166	471
Southern(LB, *hpt* probe)	93	96	188
Southern (RB, *gus* probe)	46	30	76
TAIL-PCR (LB)	34	28	62
extragenic	13	15	28
TAIL-PCR (RB)	10	6	16
near ATG start	4	1	5
near centromere	3	1	4
Target lines	3	4	7

Table S2. Primers used for this study

Name of primers	Sequence (5’ - 3’)	Function
hptF	tcgtccatcacagtttgcc	qRT-PCR for *hpt*
hptR	tcggtcaatacactacatggc	qRT-PCR for *hpt*
spsF	ttgcgcctgaacggatat	qRT-PCR for *SPS*
spsR	cggttgatcttttcgggatg	qRT-PCR for SPS
gusplusF:	cgtcccaagcagttacaatg	PCR and qRT-PCR for *gusplus*
gusplusR	cgttcgtaccagacatatccg	PCR and qRT-PCR for *gusplus*
LAD1	acgatggactccagagcggccgc(g/c/a)n(g/c/a)nnnggaa	Primers for TAIL-PCR for target line T-DNA insertion site analysis
LAD2	acgatggactccagagcggccgc(g/c/t)n(g/c/t)nnnggtt	
LAD3	acgatggactccagagcggccgc(g/c/a)(g/c/a)n(g/c/a)nnnccaa	
LAD4	acgatggactccagagcggccgc(g/c/t)(g/c/t)n(g/c/t)nnncggt	
AC	acgatggactccagag	
RB1	cgtgactgggaaaaccctggcgtt-	RB specific primers for transformants of pZH36 and pZH37 transformants
RB2	acgatggactccagtccggcccaacttaatcgccttgcagcacatc	
RB3	gaagaggcccgcaccgatcgccctt	
LB1	ggatcgccacgactccgggcgta	LB specific primers for transformants of pZH36 and pZH37
LB2	acgatggactccagtccggccgtcgggcgtacacaaatcgcccgc	
LB3	ccgagggcaaagaaatagccatggg	
hptpF	atgaaaaagcctgaactcaccgcgac	*hpt* probe for Southern blot
hptpR	ctatttctttgccctcggacgagtgc	
gusplusрF	atggtagatctgagggtaaatttcta	*gusplus* probe for Southern blot
gusplusрR	tcacacgtgatggtgatggtgatggc	
gfppF	atggtgagcaagggcgaggagctg	*gfp* probe for Southern blot
gfppR	ttacttgtacagctcgtccatgc	
barpF	atgagcccagaacgacgccc	*bar* probe for Southern blot
barpR	tcaaatctcggtgacgggca	
a	cagattgcttggctatggct	Recombination junction for type I site-specific integration
b	ggtgaactaccgatgatcgtaaga	
c	cggcgaactaataacgctca	
d	tcaaatctcggtgacgggca	
e	accgcctttgagtgagctga	Recombination junction for type II site-specific integration
f	tgatccaagctcaagctgct	
g	tcaaatctcggtgacgggca	
h	cggcgaactaataacgctca	
Bxb1-f	atgagagccctggtagtcatcc	Bxb1 integrase
Bxb1-R	cgacatcccggtgtgtagc	
